# Nontoxigenic *Vibrio cholerae* Septicemia in an Immunocompromised Patient

**DOI:** 10.1155/2012/698746

**Published:** 2012-07-10

**Authors:** Kamran Kadkhoda, Heather Adam, Matthew W. Gilmour, Gregory W. Hammond

**Affiliations:** ^1^Cadham Provincial Public Health Laboratory, Winnipeg, MB, Canada; ^2^Department of Medical Microbiology and Infectious Diseases, University of Manitoba, Winnipeg, MB, Canada; ^3^Diagnostic Services of Manitoba, Winnipeg, MB, Canada; ^4^National Microbiology Laboratory, Public Health Agency of Canada, Winnipeg, MB, Canada R3E 3R2

## Abstract

We report a recent case of non-O1, non-O139, nontoxigenic *Vibrio cholerae* septicemia in a post-liver-transplant immunocompromised patient associated with prior seafood consumption. Non-O1, non-O139 *V. cholerae* strains have been reported in several cases of extraintestinal infections and seem to be emerging infectious agents especially in patients with immunocompromising conditions.

## 1. Case History

On June 16, 2011, a 21-year-old female presented to the emergency department with a short duration of fever, chills, abdominal pain, and tachycardia. She denied having a recent history of diarrhea. On physical examination, generalized abdominal tenderness was present, but there were no signs of peritoneal irritation. Blood and urine cultures were obtained, along with blood specimens taken for routine hematology and biochemistry tests. Total bilirubin was 25.7 *μ*mol/L (normal 2–20 *μ*mol/L), GGT was 78 U/L (normal 5–29), and INR was 1.6 (normal 0.9–1.2). She was given intravenous piperacillin/tazobactam 3.375 gram along with hydration and improved rapidly. She was discharged without antibiotics. By June 20, 2011, her blood culture had been reported positive for an unidentified aerobic Gram-negative organism.

The patient had no travel history for over a year but had a complex past medical history, which included biliary atresia that culminated in an autologous liver transplant in 1996 when she was 6 years old. She was receiving Sirolimus 3 mg/day and in addition took ferrous sulfate and vitamin D 1000 IU/day. Her recent history was that on March 6, 2011, she had presented to the same emergency department with fever (39.5°C), abdominal pain, nausea and vomiting, but no diarrhea. She was found to have an *Escherichia coli* bacteremia, thought to have resulted from a liver abscess found by CT scan. She also had splenomegaly by ultrasound. She was treated with intravenous ceftriaxone 2 g/day until the liver abscess had been resolved by ultrasound. Her WBC count at that admission was 1.5 × 10^9^/L (normal 4.5–11.0), neutrophil count was 2.9 × 10^9^/L (normal 2–7 × 10^9^/L), platelets were 46 × 10^9^/L (normal 140–440), and haemoglobin was 99 g/L (normal 120–160).

## 2. The Study

A hemolytic, non-lactose-fermenting Gram-negative bacilli was isolated from a positive blood culture on a BacT/ALERT 3D instrument. The organism was preliminarily identified as *Vibrio cholerae* by an API 20E (BioMerieux) and referred to the Health Sciences Centre, Winnipeg, MB, Canada (the reference laboratory for Diagnostic Services of Manitoba rural sites) for identification and susceptibility testing. The organism was identified by VITEK 2 (bioMerieux) as *Vibrio cholerae* and was reported to the clinician as *Vibrio* spp. with appropriate antimicrobial susceptibility testing (AST) results as it is a rarely identified organism. The isolate was referred to Cadham Provincial Public Health Laboratory for further identification and to conform to the Regulations of the Manitoba Public Health Act, which includes *V. cholerae* as a notifiable organism.

As a reference isolate with previously known presumptive identification, the isolate was subcultured onto sheep blood agar, MacConkey agar, and TCBS media and incubated overnight at 35°C in an ambient air incubator. On blood agar, *β*-hemolytic colonies appeared overnight, that on Gram stain were curved Gram-negative bacilli and were oxidase positive. There were also non-lactose-fermenting colorless colonies on MacConkey agar and yellow-colored colonies on TCBS. The isolate was identified as *Vibrio cholerae* with 94.42% probability using VITEK 2 instrument. Initial 16S rDNA sequencing using a BLAST search of GenBank (NCBI, NIH, Bethesda, MD, USA) showed 99% probability for both *Vibrio cholerae* and *Vibrio mimicus*, but based on the presence of yellow colonies on TCBS, the isolate was identified as *Vibrio cholerae*. The isolate tested negative for beta-lactamase using the phenotypic method. The AST was done using the VITEK 2 instrument, disk diffusion method, and E-test (bioMerieux) and the isolate tested susceptible to ampicillin, azithromycin, trimethoprim-sulfamethoxazole, and tetracycline based on the CLSI criteria indicated in document M45-A2. The isolate was forwarded for confirmation to the Vibrio Reference Service (VRS) at the National Microbiology Laboratory (NML) in Winnipeg, MB, Canada.

At the NML, mass-spectrometry-based identification was completed using the Bruker Autoflex III according to the manufacturer's instructions. The isolate's spectra were analyzed against the manufacturer's entire taxonomy database and matched with *V. cholerae* with a score of 2.312 (correlated to “highly probable species identification”). Additionally, against a custom database of 16 reference *V. cholerae* isolates, this isolate scored 2.507, further substantiating the highly probable species-level identification as *V. cholerae*. A *Vibrio*-specific molecular speciation assay [1] was also used to confirm the identity of this isolate. Amplification and DNA sequencing of the *ftsZ *allele using oligonucleotides ftsZF1 and ftsZR2 for a 1041 bp fragment demonstrated 100% nucleotide identity between this isolate and reference non-O1 *V. cholerae *strain ATCC 35971, and 99.7% nucleotide identity between this isolate and *V. cholerae *O1 reference strains ATCC 31503 and NH-V-86. Antisera produced at the NML for O1 and O139 serogroups were used in the standard slide agglutination method and the isolate serogrouped as *V. cholerae* non-O1, non-O139.

The isolate was also positive for both *hly* and *rtx* genes, which is typical for most *V. cholerae* strains but tested negative for *ctx* gene using primers *ctx2* and *ctx3* [2] under the conditions described by Clark et al. [3].

After receiving follow-up notification and enquiries by public health, the patient was seen by an infectious diseases specialist. She had remained well with no signs or symptoms of infection since recovering from her febrile illness of the septicemia due to the liver abscess in March of 2011, until the June 2011 illness. She and her mother revealed that the patient had a fondness for shrimp and had cleaned, cooked, and eaten a meal of shrimp a few days before the onset of her most recent illness in June of 2011. There was no other history of seafood exposure and no opportunity to sample the presumptive contaminated shrimp. As an immunocompromised individual, she was treated with a 2-week course of amoxicillin. Stool cultures in the follow-up period were negative for *Vibrio* organisms.

## 3. Conclusions

Following *Vibrio parahaemolyticus* and *V. vulnificus*, the non-O1 and non-O139 serogroups of *V. cholerae* are the most commonly isolated *Vibrio *from extraintestinal sites in North America. Whereas the O1 and O139 serogroups are typically toxigenic and cause classical cholera, the other serogroups are often nontoxigenic and tend to cause extraintestinal infections such as wound infections, external ear infection, sepsis, epidural and subdural abbesses. From 1997 to 2011, amongst the total 117 human clinical non-O1/non-O139 isolates of *V. cholerae* that were submitted to the NML for identification, 61 (52%) were isolated from stool, 24 (29%) where from unknown sources, 16 (14%) from ear infections, 3 (0.03%) from urine, 2 wound isolates (0.02%), and notably the only blood isolate is described in the study herein.

Risk factors for bacteremia include underlying liver disease and malignancies, as exemplified by the case presented here. This patient was also immunocompromised, putting her at greater risk. The case fatality rate in these patient populations can be as high as 65% [4, 5]. Similar to this individual's case which was associated with the recent consumption of shrimp, other *Vibrio* infections are associated with the consumption of sea foods of any kind [4, 6, 7]. Amongst the non-O1, and non-O139 serogroups that have caused sporadic cases of diarrhea in the United States, O75 and O141 have been observed, although both harboured toxin [8, 9].

If *V. cholerae *(either toxigenic or non-toxigenic) emerges as a common contaminant of widely distributed or widely consumed food types, this will require capacity for prompt and correct diagnosis and AST as well as public health notification and investigation for effective patient management and infection control. This demands an integrated effort involving specialized reference laboratories such as the VRS in Canada.
